# Psychological Barriers to Digital Living in Older Adults: Computer Anxiety as Predictive Mechanism for Technophobia

**DOI:** 10.3390/bs9090096

**Published:** 2019-09-11

**Authors:** Dina Di Giacomo, Jessica Ranieri, Meny D’Amico, Federica Guerra, Domenico Passafiume

**Affiliations:** Department of Life, Health and Environmental Sciences, University of L’Aquila, 67100 L’Aquila, Italy

**Keywords:** digital skills, computer anxiety, adulthood use of technology, aging

## Abstract

This study aimed to investigate the emotional impact of technology use in an Italian adult population and to detect technophobia. This cross-sectional study was conducted with 117 Italian participants (age range of 50–67 years). Measured variables were computer anxiety and technology use ability. The results revealed technophobia features in the Italian adult population related to inadequate management of technology. One-way analysis of variance and Bonferroni’s post-hoc analysis showed that non-autonomous (*p* < 0.01), low-frequency (*p* < 0.01), and feeling-a-need-for-help users (*p* < 0.01) had higher levels of computer anxiety. Based on our data, although lifelong learning is a powerful digital need, a considerable proportion of the adult population is not digitally skilled, enlarging the gap between young (native digital) and adult (digital and non-digital adults and seniors) populations. Adult inclusivity in digital living is inadequate and likely affects their quality of life. Thus, our findings highlight technophobia as a possible new risk factor for Italian adults because it can affect their daily life through low adherence to digital living; rather than aging successfully, they could develop fragile aging.

## 1. Introduction

Societies and economies are experiencing significant digital and technological changes. Digital skills are now as vital as literacy, and the world needs digitally competent people who are able to not only use but also innovate and lead in using these technologies [1]. Existing literature indicates that some people may experience difficulties and frustration with technologies owing to both cognitive and psychological influences. Although the benefits of computer learning and applications are abundant, evidence on the negative side has not been sufficiently investigated. 

Computer anxiety is “concept-specific and covers a wide variety of situations in which people interact with computers” [2] (p. 711). The late adoption of technology and related products and services is a trend mostly observed in people who have misconceptions about technology, are technophobic, or lack information technology literacy [3]. Technophobia, which is not an officially recognized mental illness, is the extreme and irrational fear of technology. Technophobia is described as “abnormal fear or anxiety about the effects of advanced technology, affecting one third of the population, causing health problems and the inability to work efficiently” [4,5]. This fear is related to an irrational fear of computers, robots, artificial intelligence, weapons, and other such objects that seem advanced in scientific thought [5].

People who feel intimidated by these objects are more likely to experience technology anxiety, and frequently, the non-tech savvy older population faces a risk of lowered quality of life and chances of inclusion [2]. The ability of older adults to use digital solutions and tools is a crucial issue, because low adaptation to the digital era is a barrier in daily living that reduces quality of life, independence, autonomy, and mental health; additionally, such tools could effectively enhance medical care in older adults [5,6]. Fear of technology is prevalent among older generations who deal with computers, complicated acronyms, or digital games; however, such fear is not seen in the case of mobile phone use, which has been attributed to socioeconomic success [7,8].

So far, little scientific attention has been paid by clinical professionals to technophobia in the Italian population. Studies on the interaction of adults with technologies are almost entirely conducted on the technical aspect of the devices and the potential improvement the devices and tools can bring to the quality of life of people, more so among elderly individuals and in fragile aging, to build a friendly and positive domestic environment for older adults in their everyday life [9,10,11,12,13]. Previous studies have investigated ATM use experience in old age [14], relating the evaluation of technophobia to agreement regarding general statements on technology; and the internet effect on digital inclusion outcomes regarding users and their views of business and internet/online skills [15,16,17,18]. A further interesting topic is the impact of technology in the cognitive development [19,20,21,22,23,24] and improvement of cognitive ability in older adults [25,26,27,28,29]. An emerging topic that, so far, has been lacking research is the impact of technology on human living with respect to digital solutions adapted to the needs of individuals and society. Aging still represents a midmost variable, and in order to better profile it, it has to be taken into account. National longitudinal surveys on ICT and computer use in Italy are available through ISTAT (the Italian National Institute of Statistics), highlighting that the use of computers and the internet in the older population is increasing since 2001, but the digital skill gap remains. In 2015, 60.4% of users in the age range of 55–59 years old, 45.9% in the age range of 60–64 years old, 25.6% in the age range of 65–74 years old, and only 6.7% of 74 years old and older have used the internet [30].

An analysis of the predictive and protective factors seems urgent to overcome the low use of assistive technology among older adults who favor better digital living.

This study aimed to define the psychological features of the Italian adult population in their interaction with technology to shed light on the influence of digital living. We tested the hypothesis that emotional traits such as anxiety may be strictly related to the reduced use of technology in elderly individuals, leading to negative outcomes in digital inclusion. In particular, we wanted to determine the impact of technophobia in the Italian adult population as an obstacle to digital living.

## 2. Materials and Methods

### 2.1. Ethics Statement

Informed consent was obtained from each participant, and the study adhered to the Declaration of Helsinki [31].

### 2.2. Participants

A total of 117 adults (n = 64 female, n = 53 male) aged 50 years and older were recruited from a metropolitan area in central Italy. The participants ranged in age from 50 to 67 years, with a mean age of 69.43 years (SD = 6.01). Table 1 reports the demographic data of the sample.

The inclusion criteria were as follows: (a) Aged 50–70 years, (b) no sign of psychiatric or neurological diseases, and (c) gave informed consent.

### 2.3. Test

The measurement was based on reported demographics, technology use, and psychological traits.

The battery was composed of two questionnaires. The first was an ad-hoc *self-report* collecting demographic (education, occupation, area of residence) and technology use data (e.g., frequency and typology of use). The second was the standardized test Short Computer Anxiety Scale (SCAS).

(a) *Technology Use Self-report (TUS).* The TUS is an ad-hoc questionnaire with two sections: (1) Demographic data and (2) technology use data. The demographic data section obtains information about the respondent’s education, occupation, and area of residence. The technology use section aims to detect the use of technology in daily life and is articulated in four items: (Q1) How do you feel about your use of technology? (a = feeling confident, b = feeling a need for help, c = feeling unable), (Q2) What is the frequency of your technology use? (a = daily, b = no more than twice per week, c = never), (Q3) Do you use technology for social networking? (a = yes, b = no), and (Q4) Do you feel autonomous while using technology? (a = yes, b = need help). Using Q1, we devised the *Self-perception of technology use index* and categorized the sample into three levels: Feeling confident, feeling a need for help, and feeling unable. Using Q2, we devised the *Frequency of use index* with three levels: High-frequency user, low-frequency user, and non-user. Using Q3, we devised the *Social use of technology index* with two levels: Social user and non-social user. Using Q4, we devised the *User index* with two levels: Autonomous user and non-autonomous user.
(b) *SCAS* [32]. This scale is a brief measure of computer-related anxiety composed of six items (and Likert-type responses). It was used to detect the confidence in using computers. Cronbach’s alpha for the scale was 0.78 in the present study.

### 2.4. Procedure

Participation in the study was voluntary, and a signed informed consent form was mandatory. Recruitment was conducted in senior clubs following a psychological and medical screening program. Participants completed the self-report questionnaires in over 20 minutes. The psychological evaluation was carried out in a dedicated quiet room. Both enrollment and testing were conducted by trained psychologists. Scoring was performed by independent clinical psychologists. The data were collected into a database elaborated by SPSS.

### 2.5. Study Design

This research was a cross-sectional, observational study of an adult population in a medical laboratory.

### 2.6. Statistical Analyses

We examined the characteristics of the sample using descriptive statistics, such as mean with standard deviation and frequencies with percentages. The sample was categorized by indices of the TUS, and the differences between these categories were assessed with the MANOVA test for quantitative variables. When the results were statistically significant, Bonferroni’s test was used for pairwise comparisons in the post-hoc analysis. A *p*-value of <0.05 was the criterion for statistical significance. The data were processed using SPSS.

## 3. Results

Data were collected and then processed by blinded data elaboration. First, we performed a one-way ANOVA comparing the age and sex variables and SCAS performance to determine significant differences; statistical elaboration found no evidence for significance. 

Table 2 reports the raw score and ANOVA analysis comparing the SCAS scores and indices of the TUS Part 2: Technology use.

One-way ANOVA highlighted significant differences in frequency of use (*p* < 0.005), user (*p* < 0.03), and self-perception of technology use (*p* < 0.001) indices. Bonferroni’s post-hoc analyses showed that non-autonomous (*p* < 0.01), low-frequency (*p* < 0.01), and feeling-a-need-for-help users (*p* < 0.01) had higher levels of computer anxiety.

Subsequently, using the median age value (58), we divided the sample into two subgroups by age—adult (age range of 50–58 years) and senior (age range of 60–68 years)—and compared the SCAS scores using one-way ANOVA; no significant difference was found.

## 4. Discussion and Conclusions

This study aimed to investigate the emotional impact of the digital age on adults’ lives. Our research examined the computer anxiety experienced by adults in the management of technology in their daily lives. Although lifelong learning was the most powerful digital need, a considerable portion of the adult population was not digitally skilled, contributing to the enlarging gap between the young generation (native digital) and adults (divided into digital and non-digital adults and seniors) [10,11]. Taking this into account, the emergent risk in older adults is that the lack of autonomy limits them in taking advantage of assistive technology or innovative solutions for an improved quality of life, including the reduction of daily barriers and/obstacles for their own needs [12]. Our findings highlighted that the low frequency of technology use contributes to reduced autonomy and even lower self-perception of technology use, which keep the older adults not digitally skilled. Based on our data, non-digital adults seemed to experience computer-related anxiety, making them feel technophobic or unconfident regarding digital solutions. In other words, their inclusivity in digital living is inadequate and likely affects their quality of life. Therefore, technophobia can be considered a new risk factor for Italian adults because it can affect the daily lives of people through low adherence to digital living. Rather than aging successfully, they could develop fragile aging. Health care research should examine computer anxiety in depth as a new kind of risk factor for emotional fragility in old age, address the appropriate evaluations and therapeutic sessions, and predict dedicated pathways. Our data further confirmed scientific literature [7] on the impact of technology in daily life. Moreover, an added value is the evidence of a new psychological trait in adulthood strongly related to modern living: computer anxiety may be a predictive factor for technophobia, which is a barrier to the acceptance and adoption of technology such as telehealth, eHealth (electronic health technologies), and mHealth (mobile health technologies), as well as wearable digital devices for physiological parameter measurement. Following the hypothesis of the study, the low level of technology use in older adults is related to the anxiety to manage digital instruments: Elders do not feel themselves confident in digital living and tend to develop dependence behavior on this kind of health and daily innovative solutions.

Our data, although preliminary, highlighted the research and clinical needs on the embodiment of technophobia as a specific psychological feature of a mental disorder that significantly reduces adherence to societal challenges. 

Future development of this research will involve extensive investigation of the detailed emotional impact of technophobia in Italian adult and senior populations, measurement of the negative outcomes, as well as formulation of innovative educational and clinical pathways. 

A limitation of the study is the short psychological evaluation; a larger measurement of emotional traits in older adults, combined with an investigation of cognitive abilities and cognitive reserves, such as occupation type and lifespan, could draw a more nuanced image of the technophobia phenomenon. Moreover, it would be interesting to outline the needs of older adults for their digital growth. 

## Figures and Tables

**Table 1:** Demographic data of participants and their distribution in technology use indexes.

	N°(%)
*Gender*	
Female	64(54.7)
Male	53(45.3)
*Education*	
Non-graduate	16(13.7)
High school	58(49.6)
Graduate	43(36.8)
*Relationship Status*	
Married/living with partner	88(75.2)
Single	12(10.3)
Divorced/widows	17(14.5)
*Occupation*	
Unemployed	7(6.0)
Employed	69(59)
Retired	37(31.6)
Housewife	4(3.4)
*Use of Technology*	
Autonomous	84()
Non-autonomous	33()
*Use Frequency*	
High frequency	105(89.7)
Low frequency	12(10.3)
*Social Use of Technology*	
Social	100(85.5)
Non-social	17(14.5)
*Self-perception of Technology Use*	
Feeling confident	105(89.7)
Feeling need for help	12(10.3)

**Table 2:** One-way analysis of variance comparing Short Computer Anxiety Scale and technology use indexes.

	χ(sd)	F	*p*
*Use of Technology*		4.8	0.03 *
Autonomous	12.5		
Non-autonomous	19.1		
*Use Frequency*		8.5	0.005 **
High frequency	13.5		
Low frequency	22.2		
*Social Use of Technology*		0.85	0.35
Social	14.3		
Non-social	14.3		
*Self-perception of Technology Use*		16.6	0.000 ***
Confident	13.3		
Feeling need for help	23.7		

χ = mean value; sd = standard deviation. *: *p* < 0.01; ** *p* < 0.001; ***: *p* < 0.0001
